# Supplementary material for: Integrated near-field thermo-photovoltaics for heat recycling
Source: Nat Commun. 2020 May 21;11:2545. doi: 10.1038/s41467-020-16197-6 (PMC7242323; doi:10.1038/s41467-020-16197-6)
Supplement: Supplementary file 1 — Supplementary Information [file 41467_2020_16197_MOESM1_ESM.pdf]

Supplementary Information for:

Integrated near-field thermo-photovoltaics for heat recycling

Bhatt et al.

Supplementary Note 1: Device schematic

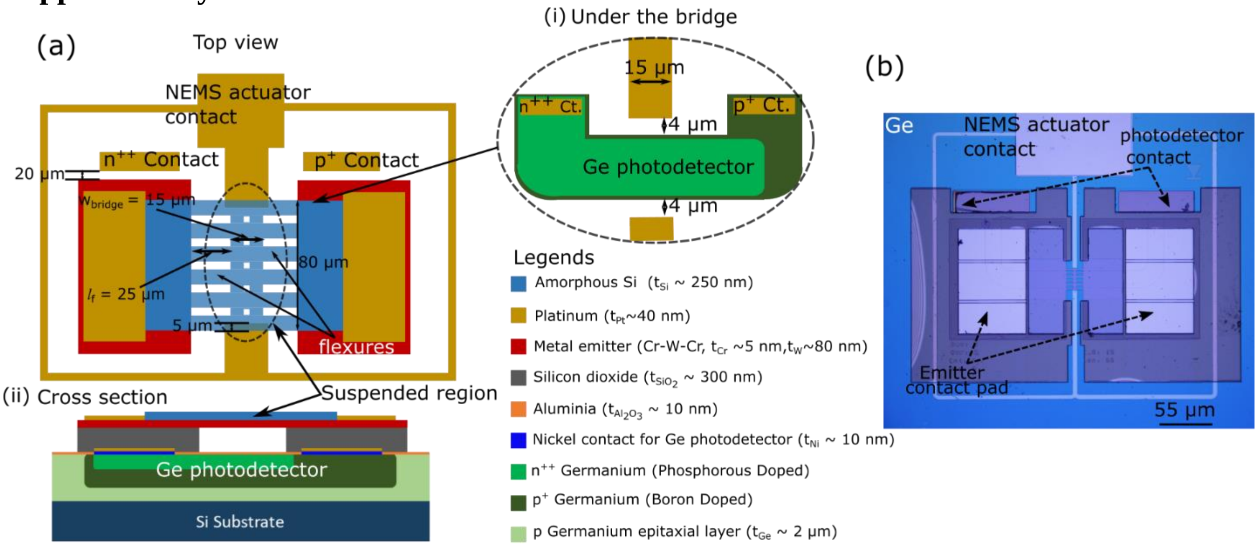

Supplementary Figure 1: Detailed schematic of the thermo-photovoltaic cell. (a) Top-view of the TPV cell showing various critical dimensions. The emitter and NEMS contact pads are ~450 μm × 450 μm. The arrangement of NEMS electrode with respect to the Ge photodetector is shown in (i) while the cross section of the TPV as seen from the NEMS contact side is shown in (ii). The color-coded legend scheme is also provided. **(b)** Microscope top view of the fabricated device showing the emitter contacts, the diode contacts, and the gate contact with gate lines running around and entering the suspended emitter area.

Supplementary Note 2: Theoretical analysis of thermo-photovoltaic

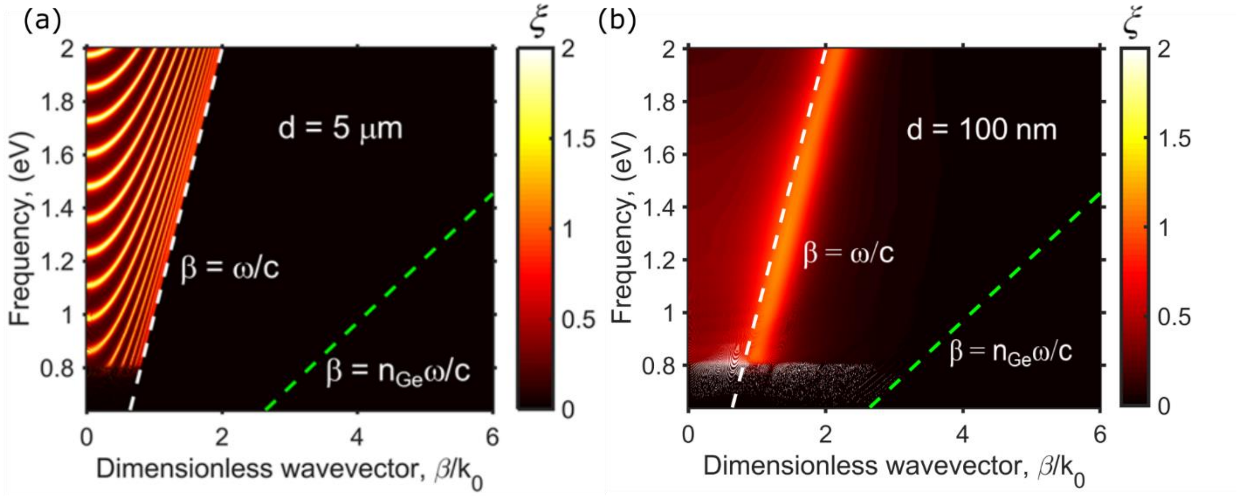

Supplementary Figure 2: Dispersion plots showing near-field enhancement effect. Energy transmission coefficient between the emitter stack and the Ge cell for different gap distances (a) d = 5 μm and (b) d = 50 nm. The dashed lines indicate the light lines of vacuum and Ge. $k_0 = \omega_0/c$ where ω_0 = 1 eV and c is the speed of light in vacuum.

The near-field radiative heat flux q is calculated based on fluctuational electrodynamics [1–4]

$$q = \frac{1}{4\pi^2}\int_0^{\infty} [\, \Theta(\omega, T_1) - \Theta(\omega, T_2)\,]\ \left[\int_0^{\infty} \xi(\omega,\beta)\beta\, d\beta\right] d\omega \qquad (1)$$

Where, Θ is the expectation value of photon energy in a single mode at angular frequency ω, β designates the magnitude of the in-plane wave vector, and ξ is the energy transmission coefficient. In Supplementary Figure 2, we show the energy transmission coefficient for both the far-field case (d = 5 μm) and near-field case (d = 50 nm). We see that, in the far field, only the propagating guided modes above the light line of vacuum are responsible for the heat transfer. In the near field, however, the high-k channels starts to mediate the radiation energy transfer through photon tunneling effect, giving rise to the enhanced power density in the current near-field TPV design.

The heat transfer simulation shown in Figure 4b in main-text is calculated considering the bending of the NEMS structure. Using the above heat transfer calculation based on fluctuational electrodynamics, the contribution of heat transfer from bending flexure of the NEMS structure is estimated and accounted for in the comparison results shown in Fig 4b of the main text. The contribution of the structural bending to heat transfer is computed using following relation:

$$P_{gen}(d) = \eta_D \cdot \left(q(d)\cdot A_{bridge} + w_F \cdot \int_{\frac{w_{bridge}}{2}}^{\frac{w_{bridge}}{2}+l_f} q\big(d'(l)\big)dl\ \right) \qquad (2)$$

Where, P_{gen} is the total generated power (Watts), q is the heat flux per unit area ($\mathrm{W\cdot m^{-2}}$), η_D is the photodetector efficiency (see Supplementary Note 7), d is the gap between the suspended center bridge of the emitter and photodetector, d' is the gap at a given point on the bridge-flexures, l is the length of bridge in microns from center towards the SiO_2 pad, w_f is the width of the flexure, l_f is the length of the flexure and, A_{bridge} is the area of the center bridge (see Supplementary Figure 1). The displacement of the bridge-flexures is simulated using FEM analysis and found to follow the given relation along its length.

$$d'(l) = -0.99 \cdot \exp\left(\left(\frac{l-0.92}{21.28}\right)^2\right) + 0.1361 \cdot \exp\left(\left(\frac{l-28.2}{11.01}\right)^2\right); \quad \frac{w_{bridge}}{2} \le l \le l_f [\mu\mathrm{m}] \qquad (3)$$

Supplementary Note 3: Surface roughness and contribution of heat transfer

The root mean squared roughness (σ) and its correlation length (τ) are measured for the emitter and detector surfaces after the Vapor-HF release step. The emitter roughness is measured on the top-Cr film, while the detector roughness is measured on the doped Ge surface (i.e. diode region under the suspended bridge), after carefully removing the top emitter bridge. The measured roughness parameters for the detector surface are σ_{Det}~ 1.1 nm and τ_{Det}~157 nm (see Supplementary Figure 3a), while those for the emitter surfaces are σ_{Emit}~1.7 nm and τ_{Emit}~54 nm (see Supplementary Figure 3b). The scattered speckles visible in the supplementary figure 3a are the residue after the vapor HF release step performed for removal of sacrificial SiO_2.

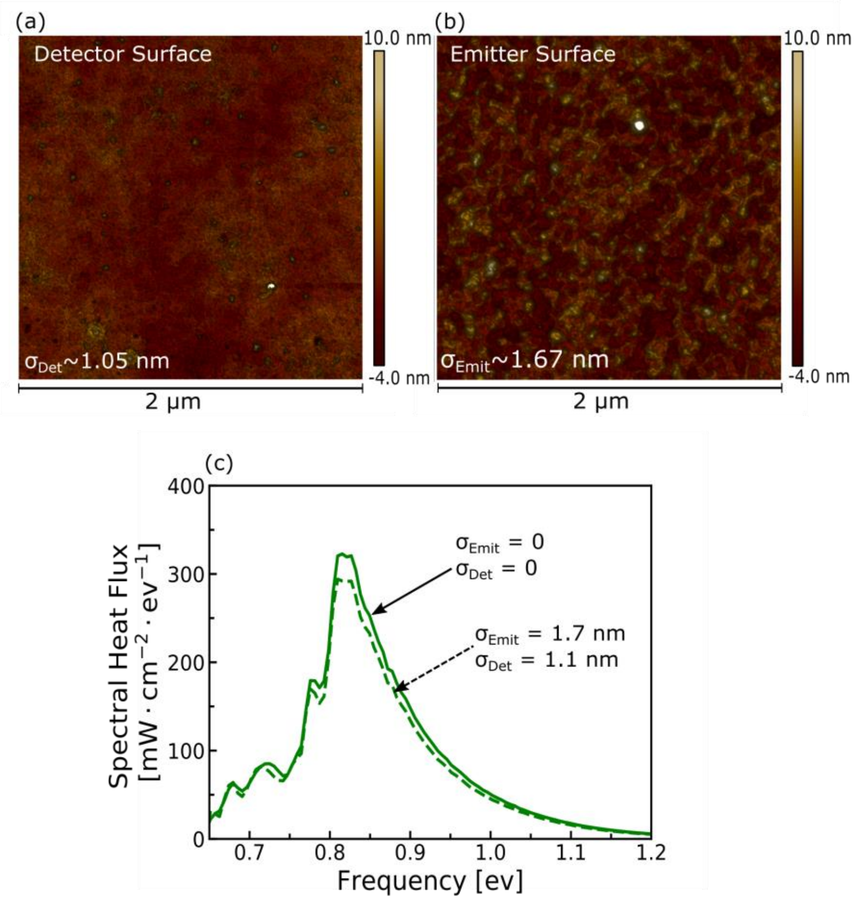

Supplementary Figure 3: Roughness of emitter and detector surfaces and, its contribution of heat transfer. Surface roughness contours of, **(a)** the detector and, **(b)** the emitter surfaces, measured using AFM. The measurement area is 4 μm^2, and σ represents the measured root mean squared roughness. **(c)** Computed spectral heat flux for the TPV stack with the emitter and detector top surfaces replaced by effective multilayer medium.

Our theoretical calculations suggest that our emitter and detector roughness induce negligible change in the spectral heat flux. The calculations are performed by splitting a rough surface into a multilayer medium [5,6]. The effective permittivity of each of this layer is computed using Maxwell-Garnet mixing formulae to estimate its effective permittivity [7]. The heat transfer is then computed as described above in Supplementary Note 2, using the multilayer model based on fluctuational electrodynamics.

Supplementary Note 4: AFM data for initial height measurement

The released TPV cells are characterized for their initial height using AFM. The AFM measurement is performed at the un-suspended contact pads to know the exact thickness of each thin films (SiO_2, Cr, W, a-Si) and then later on the central suspended region to know the actual distance from the photodetector surface (see Supplementary Figure 4). The actual gap between photodetector surface and suspended bridge is found to be 500 nm – more than the thickness of sacrificial layer due to stress from various films.

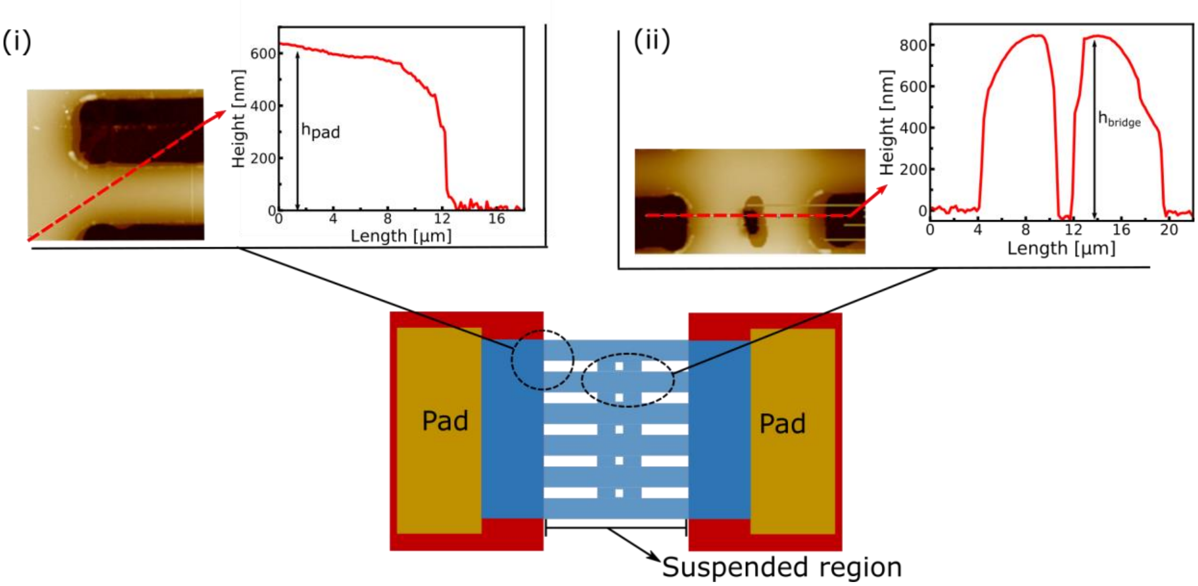

Supplementary Figure 4: Initial height measurement of the suspended bridge. A scheme of center bridge region showing locations of height measurement performed using AFM. The measurement plot shown in (i) is performed at the edge of the un-suspended pad showing a height $h_{pad} \sim 620$ nm, where $h_{pad} = t_{SiO_2} + t_{Cr} + t_W + t_{Cr} + t_{aSi}$. The measurement plot shown in (ii) is performed at the suspended bridge showing the height $h_{bridge} \sim 820$ nm. The increased height of the suspended region is due to film stress, $h_{bridge} = h_{pad} + h_{buckling}$.

Supplementary Note 5: Estimation of diode temperature

The temperature of diode is estimated by fitting the measured IV characteristics of the photodetector at cold and hot heater conditions, to the non-ideal diode model mentioned in Supplementary Equation 4. We find that the diode temperature has changed by $\Delta T_D \sim 17\ K$, when the heater temperature is maintained at $T_E \sim 880$ K $\pm$ 50 K. We attribute this change in temperature due to parasitic conduction paths as described by the thermal circuit (see supplementary figure 5).

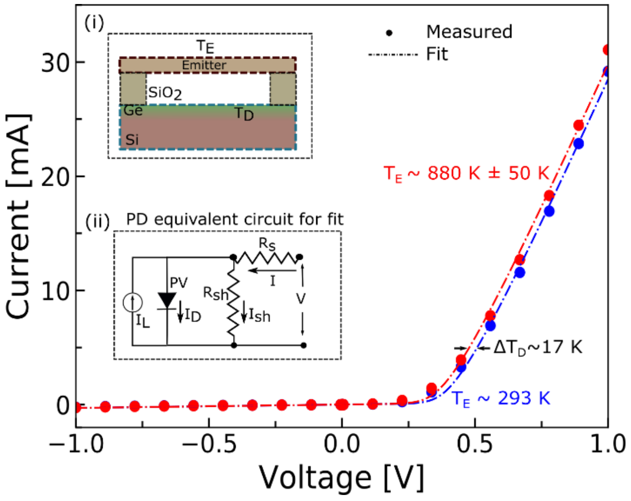

Supplementary Figure 5: Characteristics of photodetector at different emitter temperatures. Measured IV characteristics of the Ge photodetector (PD) for a cold emitter ($T_E \sim 293$ K) and a hot emitter ($T_E \sim 880$ K $\pm$ 50 K) conditions (scatter points) as shown in inset (i) and non-ideal diode model fit shown by broken line. The slight deviation between the hot and cold emitter case is suggestive of change in diode temperature that is estimated using a non-ideal diode model shown in inset (ii). The change in diode

temperature when the suspended emitter is maintained at 880 K $\pm$ 50 K is found to be ~309 K, only slightly more than the room temperature due to parasitic conduction.

Supplementary figure 5 shows the diode temperature estimated by fitting the diode IV characteristic to the non-ideal diode model as described in Supplementary Equation 4 [8–11].

$$I = -I_L + I_0 \cdot exp\left(q \cdot \frac{(V - I \cdot R_S)}{n \cdot k \cdot T}\right) + \left(\frac{V - I \cdot R_S}{R_{sh}}\right) \quad (4)$$

$$I_0 \cong A \cdot T^3 \cdot exp\left(-\frac{E_g}{kT}\right) \quad (5)$$

Where, I_L is the photocurrent, I_0 is the reverse saturation current, R_S and R_{sh} are the series and shunt parasitic resistances, T is the temperature of the photodiode, E_g is the semiconductor bandgap, V is the applied voltage across the diode and I is the measured current through the diode. k is the Boltzmann constant, n is the ideality factor and A is a proportionality constant. For fit purpose, we assume photocurrent $I_L \sim 0$, and obtain the following fit parameters: $A \sim 1 \times 10^{-4} K^{-3}$, $R_s \sim 18.5\ \Omega$, $R_{sh} \sim 3414\ \Omega$, n ~ 1.24. All the diode curves shown in the main text are zoomed-in to the power generating region of the IV plot.

Supplementary Note 6: Equivalent thermal circuit of the structure

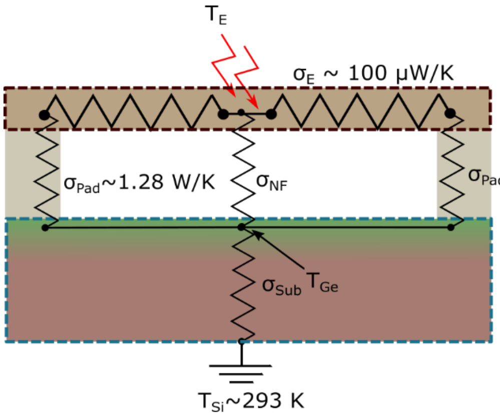

Supplementary Figure 6: Thermal equivalent circuit of the TPV cell. Thermal equivalent circuit of our TPV cell. The thermal conductance (σ) of emitter-bridge is estimated based on the experimental heating power applied while the thermal conductance of the silicon dioxide is based on temperature estimation of the photodetector and the theoretical conductivity values of SiO_2.

The heat-map shown in Fig 1c of main-text, incorporates the resulting heating due to near-field heat transfer. In the finite element heat transfer model the gap between the suspended emitter and the underlying detector is modeled as a material with thermal conductivity $k_{gap} \sim d \cdot q_{NF}/(A \cdot \Delta T)$. Where, d is the emitter-detector gap (~100 nm), A is the emitter-detector overlap area, q_{NF} is the total heat flux above detector bandgap when emitter is at 900 K and, ΔT is the temperature difference.

Supplementary Note 7: Emitter and NEMS characterization procedure

I. Estimation of Emitter Temperature

The temperature coefficient of the emitter is measured independently on multiple TPV devices on the same wafer before suspending the bridge-emitters. These device are first annealed in a vacuum chamber for a duration of 4 hours at ~550 °C. The chips are then tested in a vacuum probe station, where heated up using a calibrated temperature-controlled sample holder and the resistance is measured at different temperatures. Proper thermal contact between the chips and the heated chuck is ensured using a thermally conductive

adhesive. The resistance is measured by taking IV sweeps at currents low enough to avoid self-heating effects. The measured data is fit to the relation shown below in Supplementary Equation 6. Typical data set of the extracted resistance (slope of IVs) as a function of temperature along with the fit is shown below in Supplementary Figure 7a.

$$R_2 = R_1 \cdot [1 + \alpha_E \cdot (T_2 - T_1)] \qquad (6)$$

Where, R_2 is the electrical resistance of the emitter at temperature T_2, while R_1 is the resistance at T_1. The estimated temperature coefficient of resistance (TCR) obtained by fitting the measured data to above equation is $\alpha_E = (1.5 \pm 0.2) \times 10^{-4}\ \mathrm{K}^{-1}$.

Estimation of R_0 (i.e. resistance at $T = T_0 = 293$ K) is performed real-time during the heating of the emitter. Un-annealed and suspended emitter chips are gradually heated up by applying electrical power. The temperature ramp-up is performed relatively faster initially but gradually reduced it to a rate of $\sim$100 ℃ per hour. The slower rate is to avoid structural damage due to thermal shock to the suspended structures and allow enough time for the resistance to stabilize through the process of self-anneal. The entire heating up process takes approximately 4 hours. We sweep the input power in a small range around the parked temperature/power and simultaneously record the resistance, to estimate the stabilized R_0 and R of the emitter. Supplementary Figure 7b shows the plot of change in electrical resistance (R) of the emitter measured as a function of input electrical power (P_{in}) recorded just before performing heat-transfer measurements reported in the main-text. The response is fit to a linear polynomial function and the resistance R_0 at $P_{in} = 0$, is estimated.

$$R = r \cdot P_{in} + R_0 \qquad (7)$$

Where, R_0~78.9, $r \sim 118.20$. The temperature of the emitter is then estimated using the measured resistance at a given input power along with R_0 and α_E.

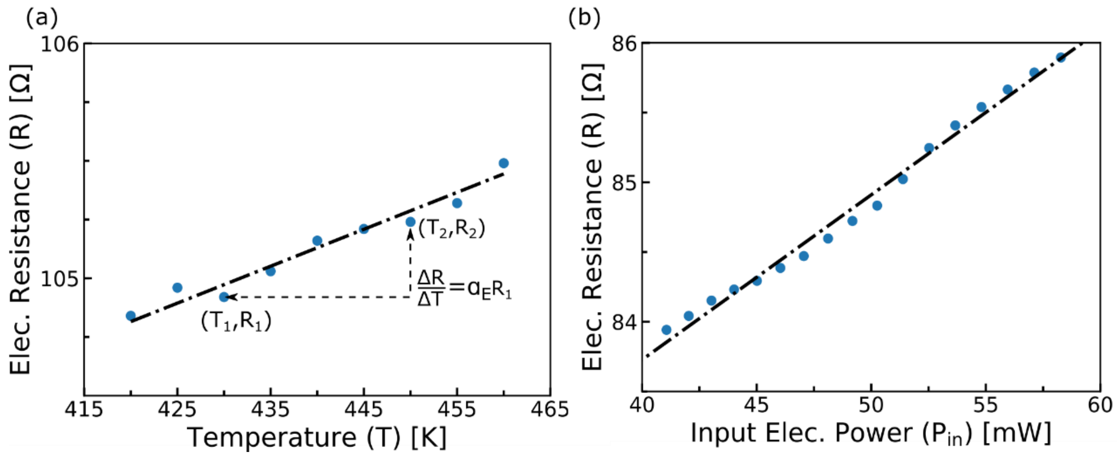

Supplementary Figure 7: Electrical characteristics of the emitter. (a) Measured resistance of the emitter for various temperatures, along with the fit and confidence-bound interval. The measurement is done for multiple TPV emitters and a typical data set is shown here. **(b)** Measured change in emitter resistance for the applied heating power. The data-set is fit to a linear polynomial to obtain $R_0(P_{in} = 0)$. The heat transfer measurements shown in main-text figure 4 are performed at P_{in}~58 mW.

II. Estimation of NEMS displacement

The characterization of NEMS is carried out using a vector network analyzer (VNA) based setup for estimation of capacitance between the suspended emitter and the underlying actuation electrode

[12–14]. We use a series-through measurement technique where the test-capacitance (DUT) is connected in a series between Port 11 and Port 21 of the VNA) and S11 parameters are measured across a frequency band (~4.6-6.6 MHz). The setup schematic is shown in Supplementary Figure 8a. The capacitance at each individual actuation voltages averaged across the chosen frequency band, and the deviation across the band is taken as the error in the distance estimation. The initial frequency, in the band is selected well above the mechanical resonant frequency of the suspended bridge (~400 kHz computed using finite element mechanics model) while the end frequency is chosen before the effects of other parasitic capacitances are observed in the S11 parameter measurement. The RF signal voltage during the measurement is set at $V_{rms} = 20$ mV. The spectral data of the recorded change in capacitance is shown in Supplementary Figure 8b. Supplementary Figure 8c shows the extracted change in capacitance for the suspended and unsuspended emitter (sacrificial SiO_2 intact). We observe ~60% change in capacitance for the suspended NEMS structure while that for the un-suspended structure is negligible, indicating that the change in capacitance is indeed due to the displacement of emitter.

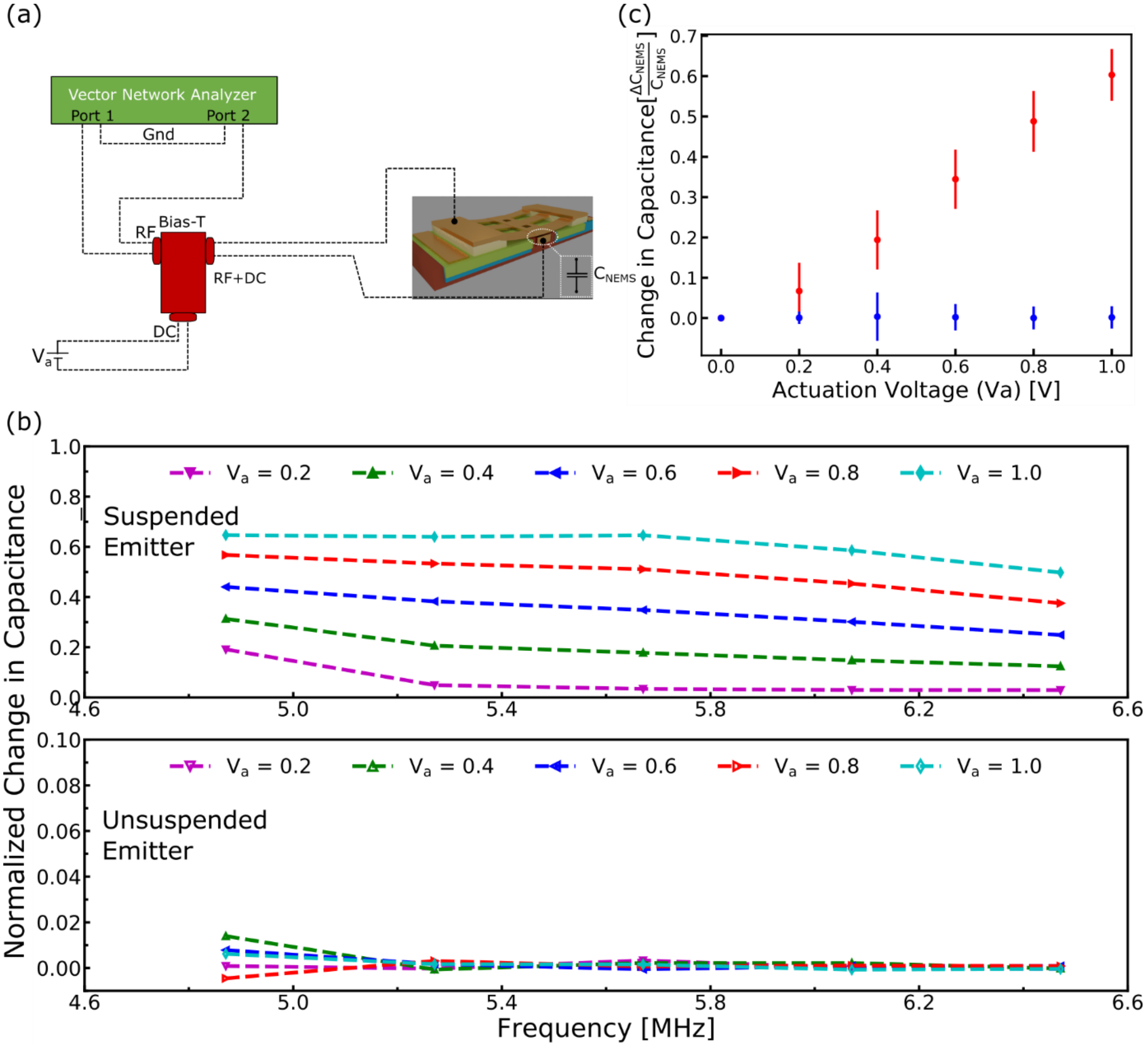

Supplementary Figure 9. Characterization of NEMS. (a) Measurement setup for estimating the change in capacitance of the NEMS structure under the influence of DC actuation potential (V_a). The gap is estimated based on the change in capacitance of the

structure and the initial estimation of gap obtained from AFM. **(b)** Change in capacitance (C_{NEMS}) of the NEMS structure as a function of applied actuation potential (V_a), for suspended and un-suspended devices. An unsuspended device has the sacrificial SiO_2 layer intact which prevents displacement of emitter thus causing the capacitance to remain unchanged.

We estimate the displacement using the change in capacitance and initial gap measurement from AFM using the following relation.

$$\delta d = -d_0 \frac{\delta C}{C} \quad (8)$$

Where, δd is the change in distance between the two capacitor plates and, δC is the resulting change in capacitance. The NEMS displacement is fit to the non-linear electrostatic actuator model given by following relation [15–17]:

$$\frac{\epsilon_0 \cdot A_{cap} \cdot V^2}{(d_0 - x)^2} \approx k_3 \cdot x^3 + k_2 \cdot x^2 + k_1 \cdot x + k_0 \quad (9)$$

Where, k_1, k_2, k_3 are the linear and non-linear spring constants while, k_0 is an arbitrary constant, ϵ_0 is the vacuum permittivity, x is the displacement, d_0 is the initial distance at $V = 0$ V, A_{cap} is the total overlap area of the actuator and the suspended emitter. The equation is simplified into following form for performing the fit.

$$V^2 \approx m_5 \cdot x^5 - m_4 \cdot x^4 + m_3 \cdot x^3 - m_2 \cdot x^2 + m_1 \cdot x + m_0 \quad (10)$$

Where, $m_5 \approx 23.9$, $m_4 \approx 26.8$, $m_3 \approx 10.5$ $m_2 \approx 0$, $m_1 \approx 0.56$, $m_0 \approx 0$ and, x is the normalized displacement. The effective displacement $\delta d = d_0 \cdot x$

III. Effect on NEMS actuation on diode characteristics

We measure the effect of the actuation potential on the photodetector characteristics at cold emitter conditions to ensure that there is no parasitic leakage between the two in-plane elements. We compare the generated current in the photodetector as a function of the NEMS potential for a hot emitter vs a cold emitter (see Supplementary Figure 9a). One can see that for the hot emitter case (i.e. emitting thermal photons), the application of actuation potential causes change in short-circuit current I_{sc} as the gap between the suspended emitter and underlying photodetector reduces. For the cold emitter case, we don't see any change in the generated current (I_{sc}) indicating that the power generated is indeed from the thermal photons and not by any other parasitic effect. The measurement of I_{sc} as a function of actuation potential (V_a) presented here is for the same device reported in the main text. We also simultaneously measure the emitter to NEMS leakage current to ensure that there is no direct short-circuit path. The measured current is shown in Supplementary Figure 9b, and found to be over 100 times smaller than the current measured at the photodetector due to collection of thermal photons.

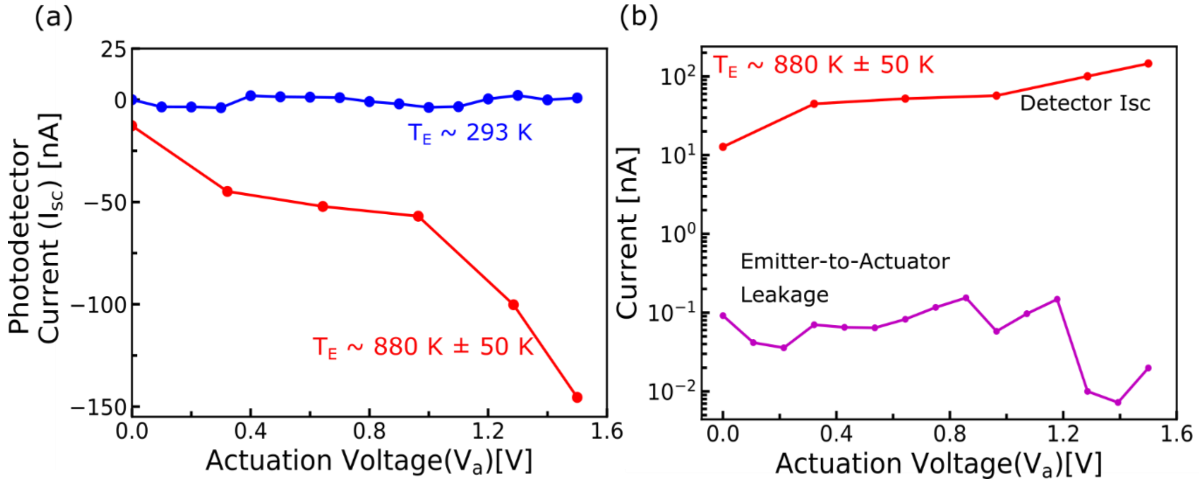

Supplementary Figure 9: Generated diode current (Isc) and electrical leakage characteristics. (a) The generated short-circuit current (I_{sc}) of the photodetector for hot (T_E~880 K ± 50 K) and cold (T_E~293 K) emitter conditions. **(b)** Generated current (I_{sc}) at the photodetector and simultaneously measured emitter-to-actuator leakage current. The leakage current between the emitter and NEMS actuator is over 100 times smaller than the current measured at the photodetector due to collection of thermal photons.

Supplementary Note 8: Photodetector response for change in polarity of emitter heating current

In order to ensure there is no current leakage from the heater to the photodetector, affecting the power generated, we measure the photodetector characteristics as we change the polarity of the bias voltage on the heater. Supplementary Figure 10 shows the measured IV characteristics of Ge photodetector for the two cases of heating current direction. The measurement is performed on an adjacent device identical to one shown in the main text. The negligible offset could be from the variation in bias potential from the instrument connected to the emitter causing change in emitter temperature.

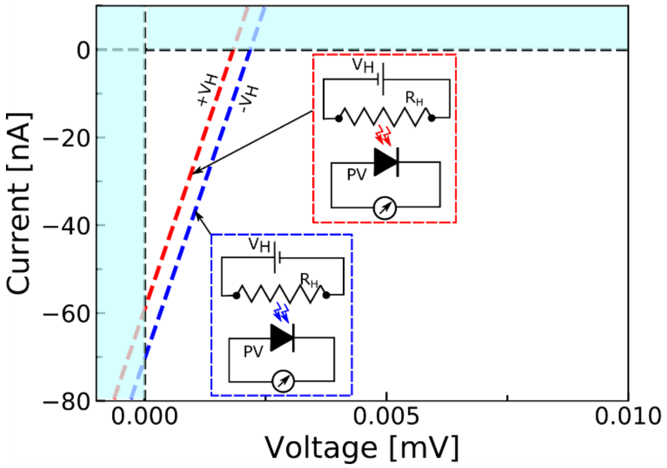

Supplementary Figure 10: Photodetector performance for inverted emitter current. Measured IV characteristics of Ge photodetector for emitter heated using positive and negative biased heater as shown in inset. The slight deviation in the IV characteristics can be due to variation in bias voltage of instrument connected to the emitter.

Supplementary Note 9: Responsivity of Ge photodetector

We measure the responsivity of our Ge photodetector cells using a broadband light source and a tunable grating filter. The light output from the source is focused on the active region of the diode using a free-space coupling setup and the resulting short circuit current (I_{sc}) and open circuit voltages (V_{oc}) are measured. Supplementary Figure 11 shows the normalized wall-plug efficiency of our Ge cell. The efficiency is normalized with respect to the power generated at λ~1500 nm ($P_{gen} \sim 357$ pW for incident optical power P_{opt}~500 μW), independently measured using a monochromatic light source. The measured diode IV characteristics zoomed-in to show power generation are provided in Supplementary Figure 11.

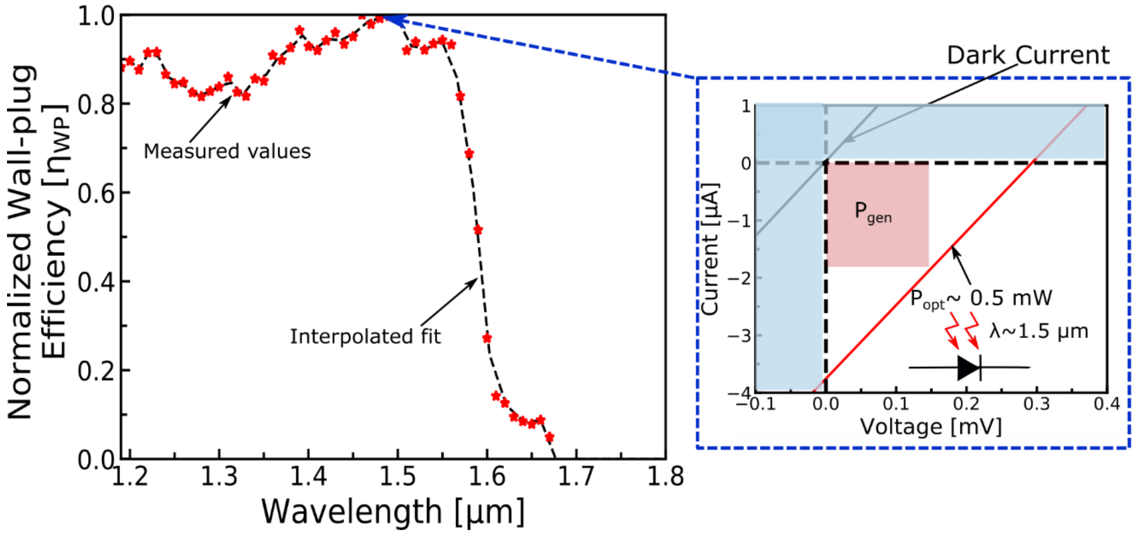

Supplementary Figure 11: Responsivity and wall-plug efficiency of Ge photodetector. Measured spectral efficiency of Ge photodetector (scatter points) performed using a broadband light source and a tunable grating filter. The plot is normalized with respect to the power generated at λ~1500 nm. Zoomed-in image of the independently measured photodetector response at λ~1500 nm using a calibrated laser source. The power generated (P_{gen}) is used to know the absolute value of spectral efficiency.

Supplementary References

1. Mulet, J.-P., Joulain, K., Carminati, R. & Greffet, J.-J. Enhanced Radiative Heat Transfer at Nanometric Distances. *Microscale Thermophys. Eng.* **6**, 209–222 (2002).
2. Zhao, B. & Zhang, Z. M. Enhanced Photon Tunneling by Surface Plasmon–Phonon Polaritons in Graphene/hBN Heterostructures. *J. Heat Transf.* **139**, 022701-022701–8 (2016).
3. Narayanaswamy, A. & Chen, G. Surface modes for near field thermophotovoltaics. *Appl. Phys. Lett.* **82**, 3544–3546 (2003).
4. Francoeur, M., Pinar Mengüç, M. & Vaillon, R. Solution of near-field thermal radiation in one-dimensional layered media using dyadic Green's functions and the scattering matrix method. *J. Quant. Spectrosc. Radiat. Transf.* **110**, 2002–2018 (2009).
5. Xu, D. Y., et al. Near-field radiative heat transfer between rough surfaces modeled using effective media with gradient distribution of dielectric function. *Int. J. Heat Mass Transf.* **142**, 118432 (2019).
6. Biehs, S.-A. & Greffet, J.-J. Near-field heat transfer between a nanoparticle and a rough surface. *Phys. Rev. B* **81**, (2010).

7. Markel, V. A. Introduction to the Maxwell Garnett approximation: tutorial. *JOSA A* **33**, 1244–1256 (2016).
8. Pierret, R. F. *Semiconductor Device Fundamentals*. (Addison-Wesley, 1996).
9. Streetman, B. G. *Solid State Electronic Devices*. (Pearson Prentice Hall, 2006).
10. Green, M. A. *Solar cells : Operating Principles, Technology, and System Applications*. (Prentice-Hall, 1982).
11. Cataldo, E., Di Lieto, A., Maccarrone, F. & Paffuti, G. Measurements and analysis of current-voltage characteristic of a pn diode for an undergraduate physics laboratory. *arXiv160805638 Phys.* (2016).
12. Stepins, D., Asmanis, G. & Asmanis, A. Measuring Capacitor Parameters Using Vector Network Analyzers. *Electronics* **18**, 29 (2014).
13. Fernández, L. J. *et al.* A capacitive RF power sensor based on MEMS technology. *J. Micromechanics Microengineering* **16**, 1099–1107 (2006).
14. Zhang, Z. & Liao, X. GaAs MMIC fabrication for the RF MEMS power sensor with both detection and non-detection states. *Sens. Actuators Phys.* **188**, 29–34 (2012).
15. Hung, E. S. & Senturia, S. D. Extending the travel range of analog-tuned electrostatic actuators. *J. Microelectromechanical Syst.* **8**, 497–505 (1999).
16. Ouakad, H. M. Electrostatic fringing-fields effects on the structural behavior of MEMS shallow arches. *Microsyst. Technol.* **24**, 1391–1399 (2018).
17. Rivlin, B., Shmulevich, S., Hotzen, I. & Elata, D. A gap-closing electrostatic actuator with a linear extended range. in *Transducers Eurosensors XXVII* 582–585 (2013).
